# Low-intensity transcranial ultrasound stimulation modulates neural activities in mice under propofol anaesthesia

**DOI:** 10.1186/s12868-023-00817-0

**Published:** 2023-08-30

**Authors:** Meiqi Liu, Yi Yuan, Xingran Wang, Teng Wang, Nannan Bian, Li Zhao, Guangying Cui, Wenchao Liu, Zhongfeng Ma, Xiaochun Yang, Shujuan Liang, Zhuo Liu

**Affiliations:** 1 Department of Anesthesiology, First Hospital of Qinhuangdao, Qinhuangdao, 066000 China; 2 School of Electrical Engineering, Yanshan University, Qinhuangdao, 066004 China; 3 Department of Thoracic Surgery, First Hospital of Qinhuangdao, Qinhuangdao, 066000 China; 4 Department of General Surgery, First Hospital of Qinhuangdao, Qinhuangdao, 066000 China

**Keywords:** Propofol anaesthesia, Low-intensity transcranial ultrasound stimulation, Local field potentials, Electromyography

## Abstract

**Background:**

Previous studies have reported that transcranial focused ultrasound stimulation can significantly decrease the time to emergence from intraperitoneal ketamine-xylazine anaesthesia in rats. However, how transcranial focused ultrasound stimulation modulates neural activity in anaesthetized rats is unclear.

**Methods:**

In this study, to answer this question, we used low-intensity transcranial ultrasound stimulation (TUS) to stimulate the brain tissue of propofol-anaesthetized mice, recorded local field potentials (LFPs) in the mouse motor cortex and electromyography (EMG) signals from the mouse neck, and analysed the emergence and recovery time, mean absolute power, relative power and entropy of local field potentials.

**Results:**

We found that the time to emergence from anaesthesia in the TUS group (20.3 ± 1.7 min) was significantly less than that in the Sham group (32 ± 2.6 min). We also found that compared with the Sham group, 20 min after low-intensity TUS during recovery from anaesthesia, (1) the absolute power of local field potentials in mice was significantly reduced in the [1–4 Hz] and [13–30 Hz] frequency bands and significantly increased in the [55–100 Hz], [100–140 Hz] and [140–200 Hz] frequency bands; (2) the relative power of local field potentials in mice was enhanced at [30–45 Hz], [100–140 Hz] and [140–200 Hz] frequency bands; (3) the entropy of local field potentials ([1-200 Hz]) was increased.

**Conclusion:**

These results demonstrate that low-intensity TUS can effectively modulate neural activities in both awake and anaesthetized mice and has a positive effect on recovery from propofol anaesthesia in mice.

## Background

Smooth and rapid recovery from general anaesthesia (GA) is the goal of anaesthesiologists [1]. Therefore, it is necessary to find a method to treat delayed recovery or delayed emergence from anaesthesia [2]. Recovery and emergence from anaesthesia involve the reestablishment of sensory, motor, and highly ordered cognitive functions and mediate general arousal by subcortical stimulation [3]. Previous studies have reported that noninvasive brain stimulation techniques, including transcranial magnetic stimulation and transcranial direct current stimulation, were used to hasten recovery from anaesthesia in animal models [1, 4–6].

Compared to transcranial magnetic stimulation and transcranial direct current stimulation, transcranial ultrasound stimulation (TUS) as a promising noninvasive neuromodulation technology [7], has the advantages of deep penetration depth and high spatial resolution [8–10]. Recently, the low-intensity TUS has been used to reversibly modulate the neuronal activity without accumulating significant thermal energy over the scalp or in the brain. low-intensity TUS induces neuronal activity in the motor cortex, which is sufficient to evoke motor behaviour. It can alter the relative power and entropy of cortical neural activities [11–14]. The effects of low-intensity TUS on excitatory and inhibitory neurons are different [15], and the neural activity caused by low-intensity TUS propagates to the surrounding area over time [16]. low-intensity TUS has also been used to locally modulate human cortical function and significantly attenuate the amplitude of somatosensory evoked potentials evoked by median nerve stimulation [17]. The above previous research results demonstrate that low-intensity TUS can effectively modulate neuronal activity both in animals and humans.

In terms of the effect of anaesthesia level on ultrasound stimulation, previous studies have shown that when ultrasound stimulation evokes motor responses in mice, ultrasound-evoked responses are rare at 0.5% isoflurane but become more frequent as the level of anaesthesia decreases [18]. Another study found that the average power of the local field potentials (LFPs) induced by low-intensity TUS in mice was significantly increased in anaesthetized and awake states, but there was no significant change in the exercise state [19]. In the anaesthetized state, low-intensity TUS enhanced phase locking between spike and ripple oscillations [19]. In addition, previous studies have reported that transcranial focused ultrasound stimulation can significantly decrease the time to emergence from anaesthesia in rats [20], however, how transcranial focused ultrasound stimulation modulates neural activity in anaesthetized rats is unclear.

To answer this question, we used low-intensity TUS to stimulate the brain tissue of propofol-anaesthetized mice, recorded the local field potentials (LFPs) in the mouse motor cortex and electromyography (EMG) signals from the mouse neck, and analysed the time to emergence from anaesthesia, mean absolute power, relative power and entropy of LFPs.

## Materials and methods

### Animal surgery

Twelve healthy BALB/c mice (all males, weighing 20–25 g, Beijing Life River Laboratory Animal Technology Co., Ltd. China) were used in this study. All procedures were performed under institutional review and approved by the Animal Ethics and Management Committee of Yanshan University.

Mice were randomly divided into two groups: the Sham group (n = 6) and the TUS group (n = 6). After anaesthetization with 2% isoflurane, the mice were fixed in a stereotaxic apparatus (68,002, 68,030, RWD Co., China), and then 1.2% isoflurane was given continuously by a face mask. Then, the fur covering the neck was shaved, and the skin was washed with 0.9% saline solution.

The skin was incised along the midline, and the anterior fontanelle, posterior fontanelle, herringbone suture and sagittal suture of the skull, subcutaneous tissue and periosteum were cleaned to facilitate ultrasound intervention. Homemade microfilament electrodes were inserted into the neck of the mice in a standard way to facilitate EMG of muscle acquisition, and a cranial drill (78,001, Warroad, China) was used to perforate the skull of the mice at the sagittal suture. Three electrodes were applied for LFPs acquisition in mice, and one microfilament electrode was inserted into the motor cortex of the brain (coordinates: anteroposterior (AP) = 1 mm, mediolateral (ML) = 1.5 mm, dorsoventral (DV) = − 1 mm); the other two electrodes were ground and reference electrodes located 5 mm above the sagittal suture in mice. After completion, electrodes were fixed using glue and dental cement.

Twenty-four hours after the surgery, the LFPs and EMG signals were recorded before the administration of propofol. Then, 70 mg/kg propofol (Diprivan, Aspen Pharma Trading Limited, Ireland) was administered by intraperitoneal injection, and the mice in the TUS group were stimulated with low-intensity TUS in the motor cortex after the administration of propofol.

### Low-intensity TUS protocol

**Fig. 1:**
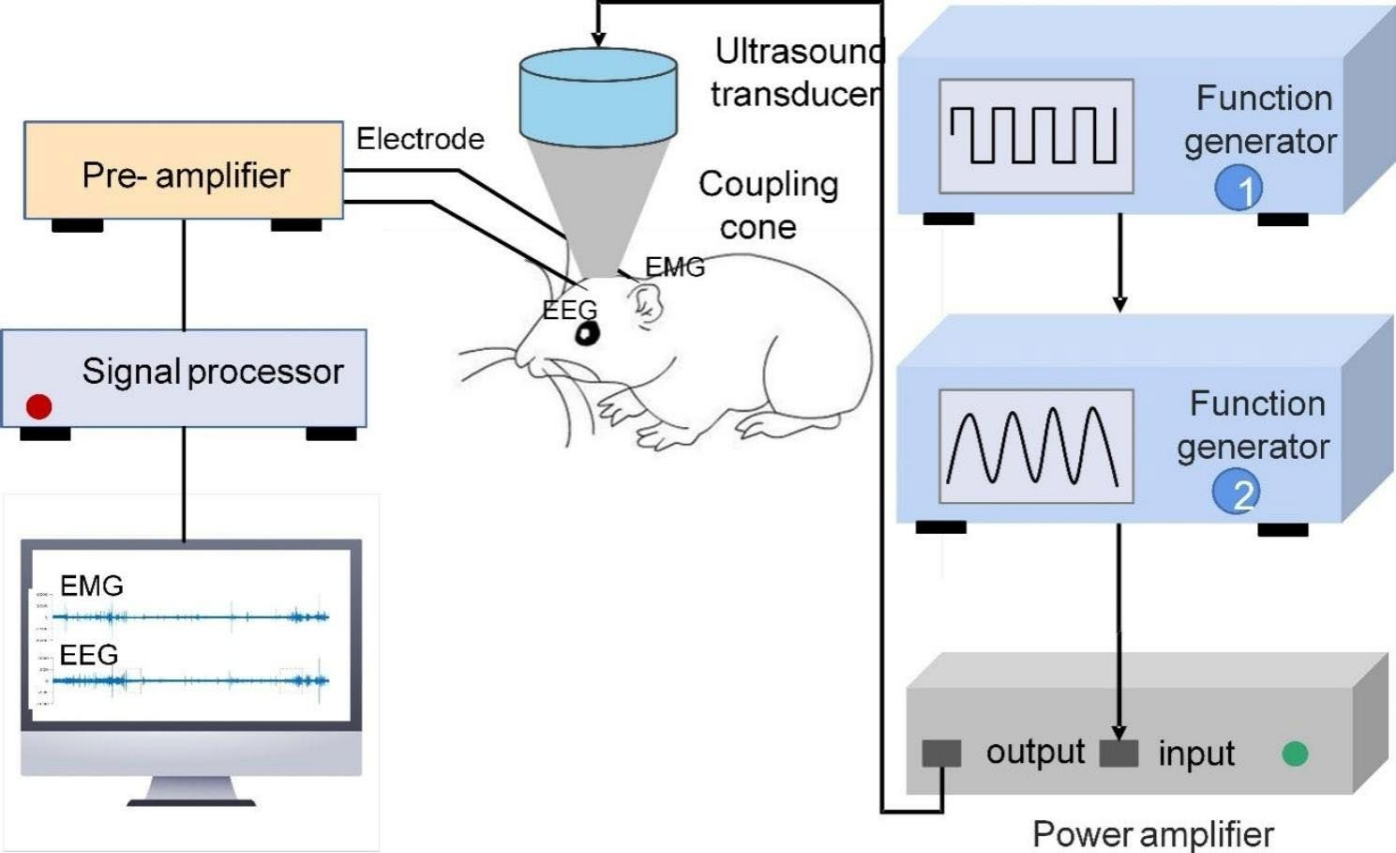
Schematic of ultrasound stimulation and EEG and EMG recording

The pulsed signal generated by two arbitrarily connected function generators (AFG3022C, Tektronix, USA) was amplified by a linear power amplifier. An unfocused ultrasound transducer with a fundamental frequency of 500 kHz (V301-SU, Olympus, USA) was used in our experiment (Fig. 1). In addition, a 3D printed conical collimator filled with US coupling gel was used to connect the mouse head with the transducer to reduce the absorption and distortion of the sound waves at a 45° angle to the electrodes. The stimulation duration, pulse repetition frequency and duty cycle were set as 50 ms, 1 Hz, and 5%, respectively. The ultrasound pressure under the skull measured by a calibrated needle-type hydrophone (HNR500; Onda, USA) in the water tank was 0.51 MPa, and its corresponding I_sppa_ and I_spta_ were 8.67 W/cm^2^ and 0.43 W/cm^2^. We determined the placement of the ultrasound transducer and coupling cone based on the mouse brain atlas and the distribution map of the ultrasound field [14]. This ensured that the ultrasound was targeted to the motor cortex. The total time for each stimulation was 5 min.

### Data acquisition

The data were sent to the neural signal processor (Cerebus Data Acquisition System, Blackrock Microsystems, USA) through a preamplifier (63,386, A-M Systems Inc., USA) and transmitted to a computer for data storage and processing.

### Power spectrum analysis, time-frequency diagram and sample entropy

We divided the data into several groups (Pre-Anes, Post-Anes, TUS-5 min/10 min/15 min/20 min). The LFPs signals one minute after the starting time of each group were used to calculate the power. The power spectrum of LFPs was analysed using the Welch algorithm [21]. We performed 1-200 Hz filtering and power frequency removal (50 Hz) processing on the LFPs data and 300–1000 Hz filtering on the EMG data. The mean absolute power was obtained and analysed from these different frequency bands of [1–4 Hz], [4–12 Hz], [13–30 Hz], [30–45 Hz], [55–100 Hz], [100–140 Hz] and [140–200 Hz]. The total absolute power of the frequency bands was obtained from the band (1-200 Hz) by summing the absolute power of the above frequency bands. The relative power of each frequency band was equal to the corresponding absolute power divided by the total absolute power. The time-frequency diagram was calculated using short-time Fourier transforms with a hamming window. The sample entropy of the signals in different frequency bands was based on the literature [22]. The sample entropy can be expressed by the following equation:

1 $$SampEn(m,r,N)=-\text{ln}\left[{C}^{m+1}\right(r)/{C}^{m}(r\left)\right]$$

where N is the length of data, m is the vector dimension, and r is the tolerance.

### Statistical analysis

SPSS 21.0 statistical software was used for statistical analysis. Continuous variables are presented as the mean ± SEM. The time to emergence from anesthesia in two groups was compared with the Mann‒Whitney test. The LFPs in the mouse motor cortex, the EMG signals from the mouse neck, the mean absolute power, relative power and entropy of LFPs at different times were evaluated with one-way ANOVA using multiple comparisons (least significant difference (LSD)). Differences were considered significant when *p* < 0.05.

## Results

### The time to emergence from anaesthesia under low-intensity TUS

**Fig. 2:**
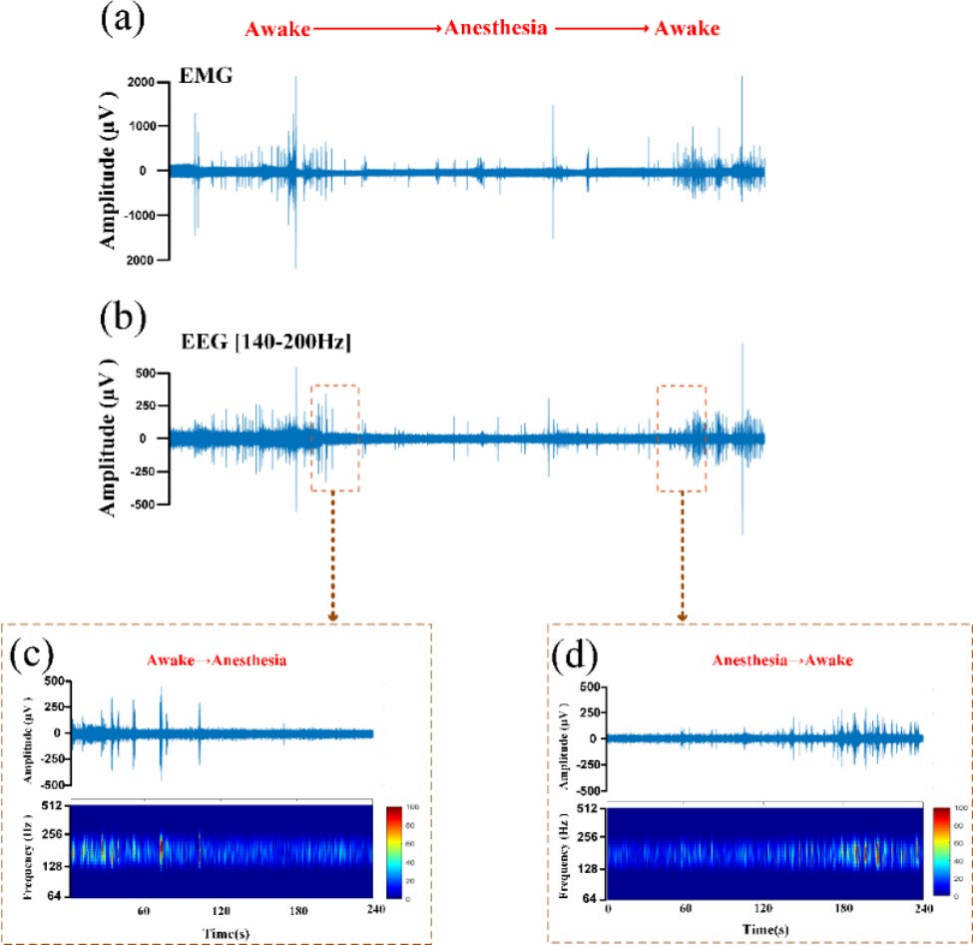
EMG and LFPs in different behavioural states (anaesthesia/awake). **(a)** A complete period (40 min) of EMG signals recorded in one mouse (40 min). **(b)** A complete period (40 min) of EEG (140–200 Hz) signals recorded in one mouse. **(c)** 4-minute LFPs signals extracted from **(b)** representing the state from awake to anaesthesia. **(d)** 4-minute LFPs signals extracted from **(b)** representing the state from anaesthesia to awake

**Fig. 3:**
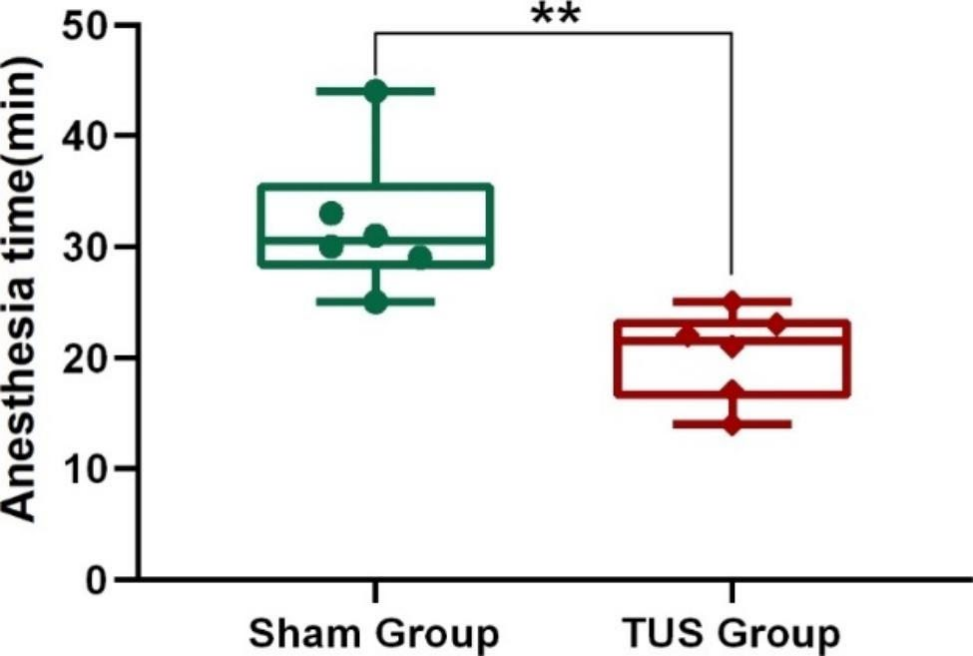
Time to emergence from anaesthesia in the sham group and TUS group (Sham group: n = 6; TUS group n = 6; means ± SEMs; **p < 0.01; Mann‒Whitney test)

First, we analysed the time to emergence from anaesthesia under low-intensity TUS. Under the same propofol dose and operation, the EMG signals changed from a disordered state to a fixed single amplitude, which suggested the beginning of the anaesthesia state (Fig. 2a). The recovery of the neuromuscular electrical signal was regarded as recovery from the anaesthesia state, which generally appeared as an intermittent change first, called the pre-wake state, and after a few minutes, an explosive change occurred. Simultaneously, the muscle strength of the mouse’s limbs was restored, the respiratory rate was accelerated, and the head could raise. This moment is defined as the time of awakening, as well as the end of the anaesthesia state. The time from anaesthesia to waking was defined as the time to emergence from anaesthesia. As shown in Fig. 2b, c and d, LFPs signals at frequency bands of 140–200 Hz showed similar changes to the EMG signal. The energy of the LFPs in the anaesthesia state was clearly less than that in the awake state. Next, we calculated the time to emergence from anaesthesia in the Sham group and TUS group, and the results shown in Fig. 3 indicate that the time to emergence from anaesthesia in the TUS group (20.3 ± 1.7 min) was significantly less than that in the Sham group (32 ± 2.6 min) (Sham group: n = 6; TUS Group n = 6; means ± SEMs; **p < 0.01; Mann‒Whitney test). The above results demonstrate that low-intensity TUS can significantly decrease the time to emergence from anaesthesia in mice, which is consistent with the results of previous studies in the literature.

### Mean absolute power of LFPs from anaesthesia to wakefulness in the sham and TUS groups

**Fig. 4:**
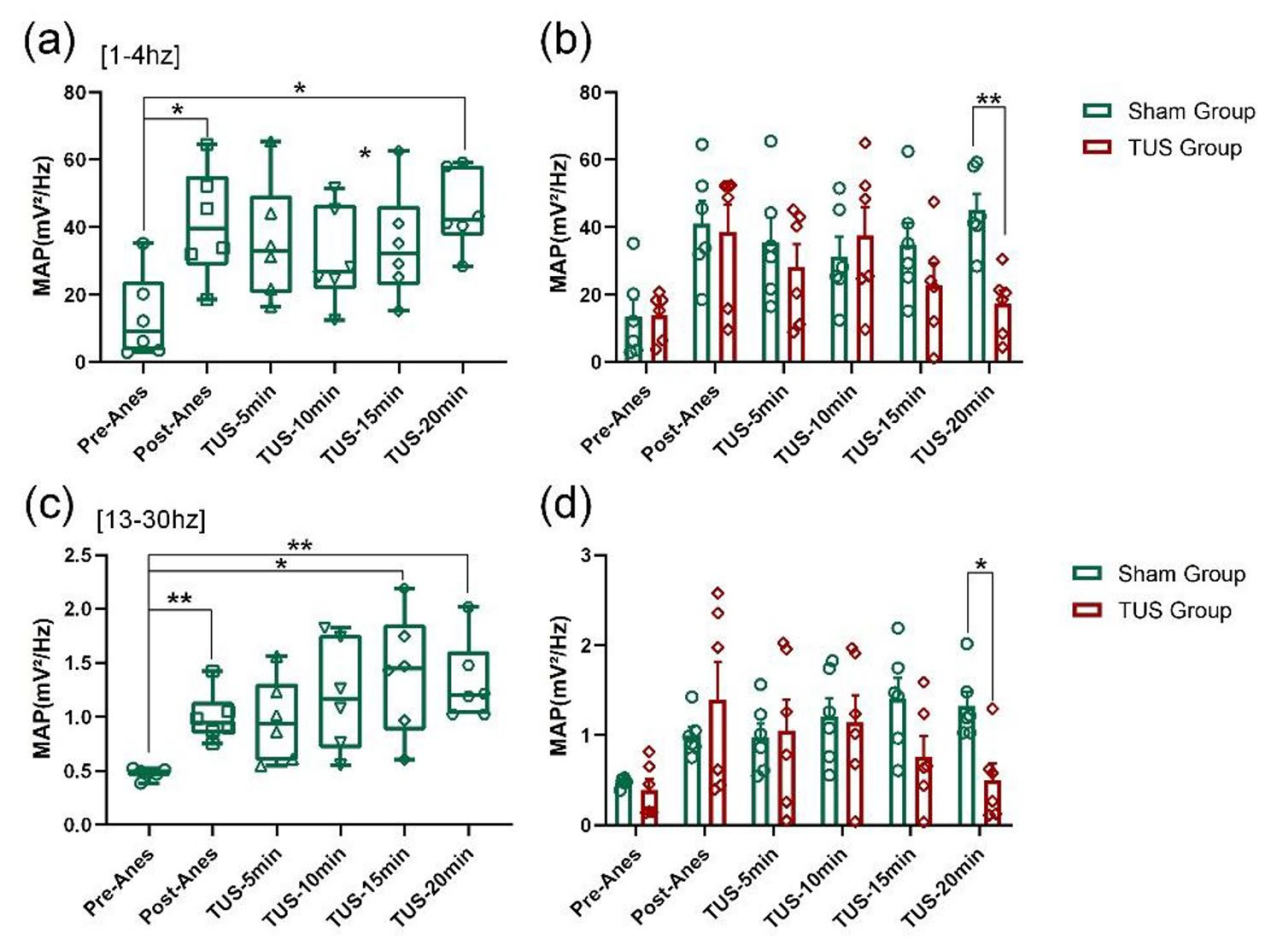
MAP of the LFPs of low frequency bands. **(a)** and **(c)** Changes in MAP at the 1–4 Hz and 13–30 Hz frequency bands, respectively, after propofol anaesthesia. **(b)** and **(d)** The MAP values at the 1–4 Hz and 13–30 Hz frequency bands in the sham group and TUS group. (Sham group: n = 6; TUS group n = 6; means ± SEMs; **p < 0.01; *p < 0.05; one-way ANOVA with multiple comparisons (LSD)).

**Fig. 5:**
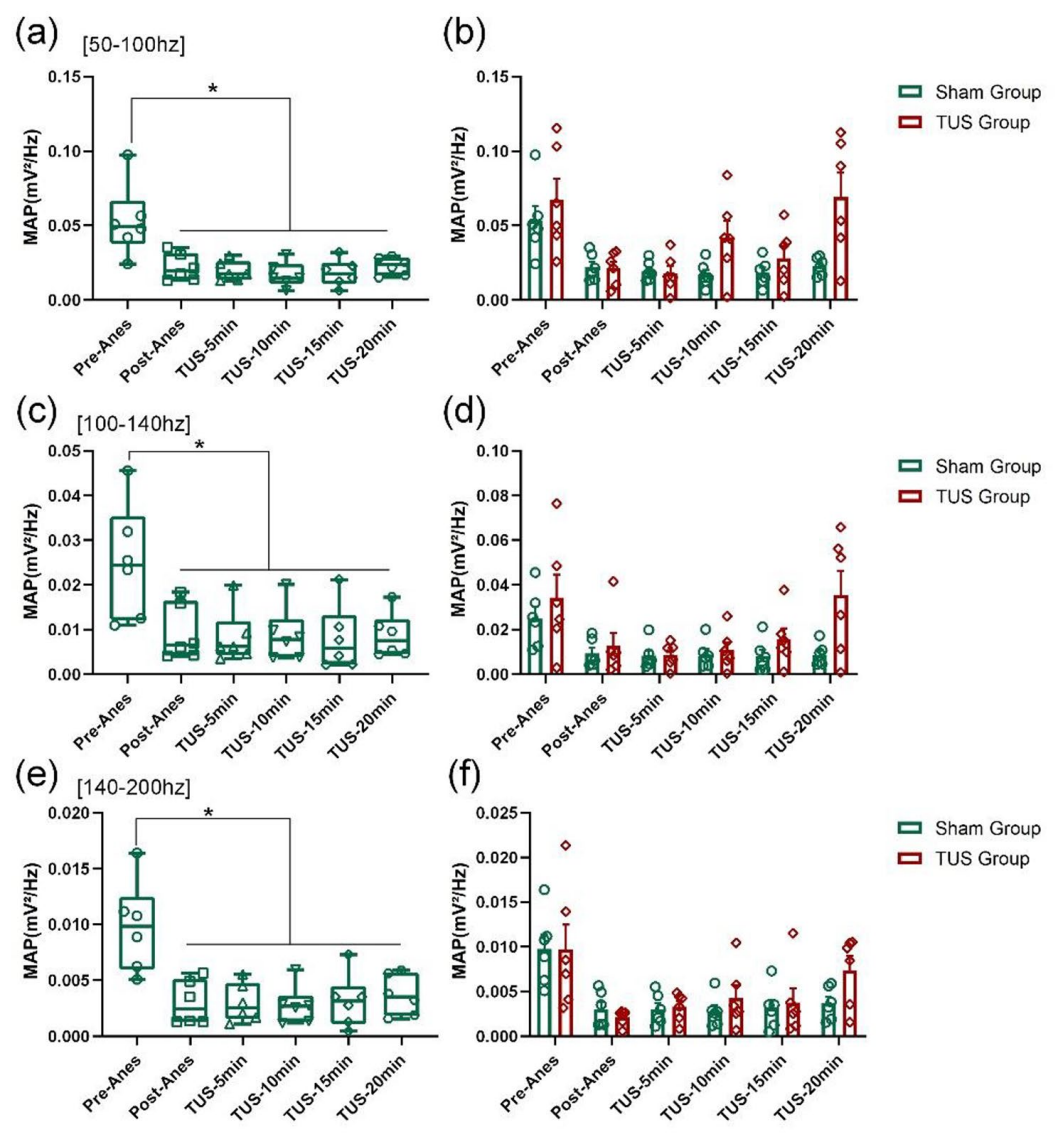
MAP of the LFPs of high frequency bands. **(a)**, **(c)** and **(e)** Changes in MAP in the 55–100 Hz, 100–140 Hz and 140–200 Hz frequency bands after propofol anaesthesia, respectively. **(b)**, **(d)** and **(f)** The MAP values at 55–100 Hz, 100–140 Hz and 140–200 Hz frequency bands in the sham group and TUS group. (Sham group: n = 6; TUS Group n = 6; means ± SEMs; **p < 0.01; *p < 0.05; one-way ANOVA with multiple comparisons (LSD)).

The power of LFPs represents a key neuromodulator parameter of neural oscillations. To evaluate the effect of low-intensity TUS on power, we analysed the mean absolute powers (MAP) and relative powers (RP) of different frequency bands in different periods of time (Pre-Anes: before propofol anaesthesia, Post-Anes: just entering into the anaesthesia state, TUS-5 min/10 min/15 min/20 min:5 min/10 min/15 min/20 min after the beginning of low-intensity TUS). We found that the absolute power of the LFPs showed an increasing trend at the 1–4 Hz and 13–30 Hz frequency bands (Fig. 4(a) and (c)) and a decreasing trend at the [55–100 Hz], [100–140 Hz] and [140–200 Hz] frequency bands with time in the TUS group (Fig. 5(a), (c) and (e)). Compared with the Sham group, the absolute power of LFPs in mice was significantly reduced at the [1–4 Hz] and [13–30 Hz] frequency bands (Fig. 4(b) and (d)) and significantly increased at the [55–100 Hz], [100–140 Hz] and [140–200 Hz] frequency bands (Fig. 5(b), (d) and (f)) at 20 min after low-intensity TUS during recovery from anaesthesia (Fig. 4(b) and (d)). (Sham group: n = 6; TUS group n = 6; means ± SEMs; **p < 0.01; *p < 0.05; one-way ANOVA with multiple comparisons (LSD)). These results demonstrate that low-intensity TUS can modulate the mean absolute power of LFPs from anaesthesia to wakefulness.

### Relative power of LFPs from anaesthesia to wakefulness in the sham and TUS groups

**Fig. 6:**
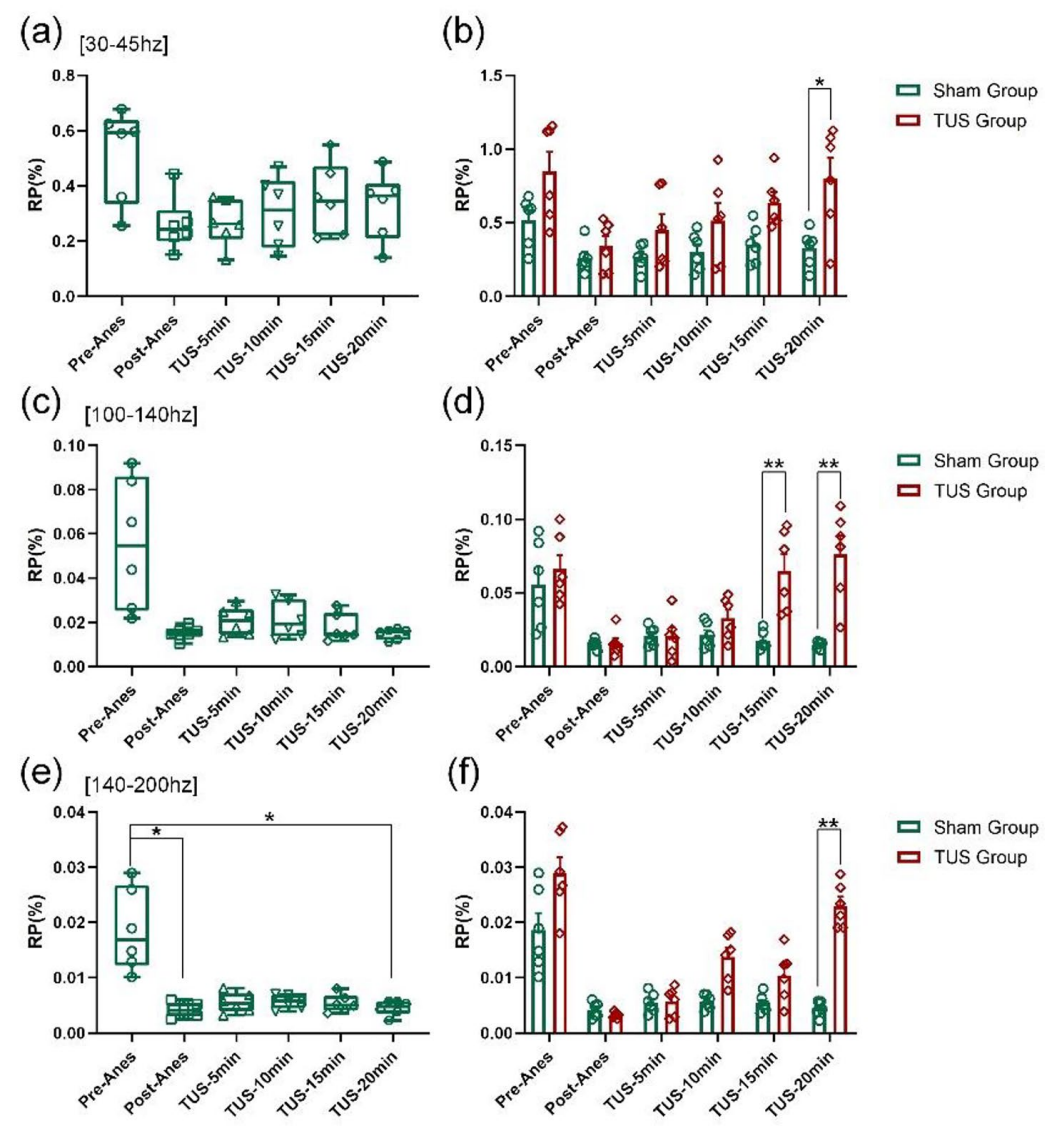
RP of the LFPs of different frequency bands. **(a)**, **(c)** and **(e)** Changes in RP of different frequency bands ([30–45 Hz], [100–140 Hz] and [140–200 Hz]) after propofol anaesthesia, respectively. **(b)**, **(d)** and **(f)** The RP values at the 30–45 Hz, 100–140 Hz and 140–200 Hz frequency bands in the sham group and TUS group. (Sham group: n = 6; TUS Group n = 6; means ± SEMs; *p < 0.05; **p < 0.01; one-way ANOVA with multiple comparisons (LSD)).

Based on the above analysis of the MAP of the LFPs, we also explored the changes in the RP of the LFPs. The results (Fig. 6 (a), (c) and (e)) showed that the relative power had a decreasing trend at the [30–45 Hz], [100–140 Hz] and [140–200 Hz] frequency bands with time in the TUS group (Fig. 6 (a), (c) and (e)). Compared with the Sham group, the RP of LFPs in mice was significantly increased at the [30–45 Hz], [100–140 Hz] and [140–200 Hz] frequency bands at 20 min after low-intensity TUS during recovery from anaesthesia (Fig. 6 (b) and (d) and (f)). (Sham group: n = 6; TUS Group n = 6; means ± SEMs; *p < 0.05; **p < 0.01; one-way ANOVA with multiple comparisons (LSD)). These results demonstrate that low-intensity TUS can modulate the relative power of LFPs from anaesthesia to wakefulness.

### Entropy of LFPs from anaesthesia to wakefulness in the sham and TUS groups

**Fig. 7:**
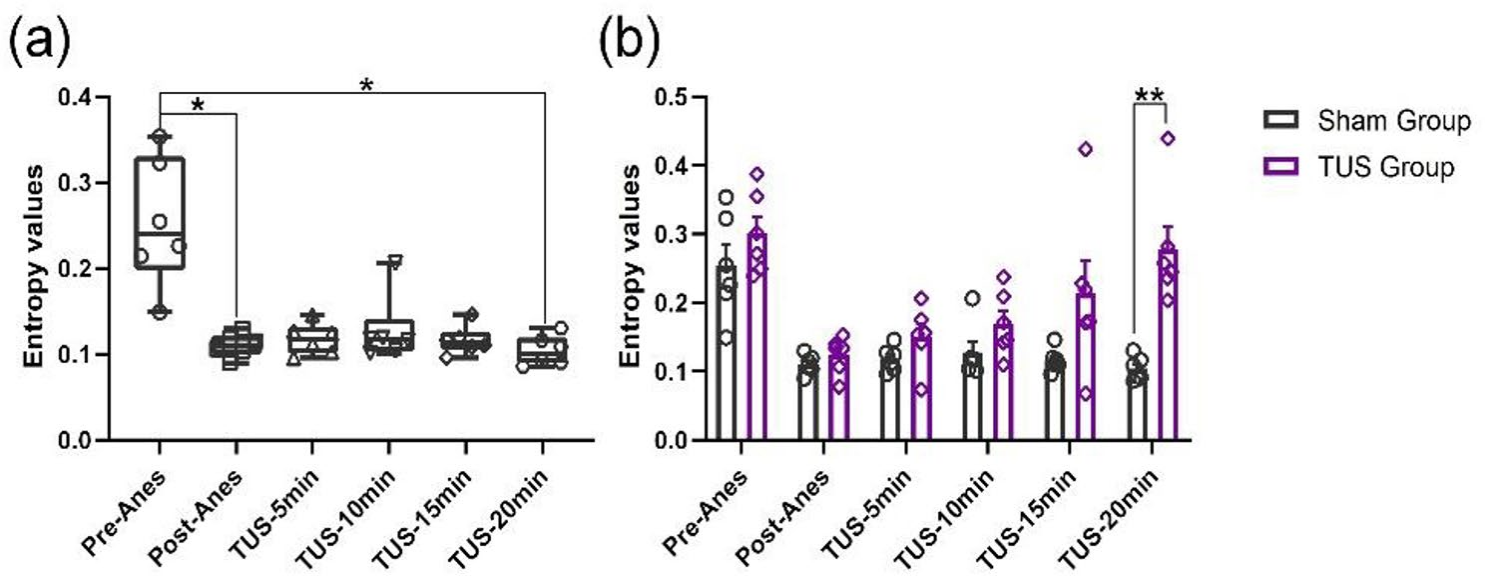
Entropy values of EEG [1-200 Hz] measured in the sham group and TUS group. **(a)** Changes in entropy in the sham group after propofol anaesthesia. (n = 6; means ± SEMs; *p < 0.05; one-way ANOVA with multiple comparisons (LSD)). **(b)** The recovery of entropy in the TUS group. (n = 12; means ± SEMs; **p < 0.01; one-way ANOVA with multiple comparisons (LSD)).

Entropy can respond to the complexity of EEG signals, serve as one of the references for neural activation, and be used as a biomarker for judging and measuring anaesthesia levels [23–25]. Figure 7 (a) shows that entropy had a decreasing trend with time in the TUS group. Compared with the sham group, the entropy of local field potentials ([1-200 Hz]) significantly increased at 20 min after low-intensity TUS during recovery from anaesthesia (Fig. 7(b)). (Sham group: n = 6; TUS Group n = 6; means ± SEMs; *p < 0.05; **p < 0.01; one-way ANOVA with multiple comparisons (LSD)). These results demonstrate that low-intensity TUS can modulate the entropy of LFPs from anaesthesia to wakefulness.

## Discussion

Neuroscientific investigations in anaesthesiology have shifted the focus from the induction and maintenance of anaesthesia to emergence and recovery from anaesthesia and highlighted that this process might be regulated under distinct neurobiological conditions and amenable to exogenous modulation [5]. low-intensity TUS as a means in neuromodulation has received considerable attention. In this study, we investigated the effects of low-intensity TUS on the emergence and recovery from propofol anaesthesia in mice, and the results showed that the administration of ultrasound stimulation can promote the awakening and neurological recovery of mice, which is consistent with the findings of Yoo et al. that ultrasound with an intensity (Isppa) of 6 W/cm^2^ significantly reduces the emergence time under ketamine and xylazine anaesthesia [20]; however, the detailed mechanism is unclear. Several hypotheses may be relevant to this process. First, low-intensity TUS may modulate the levels of neurotransmitters. Propofol produces unconsciousness partly by augmenting the effect of the γ-aminobutyric acid (GABA) receptor [26]. GABA-mediated inhibition of neuronal network activity underlies both sleep onset and maintenance [27–29]. low-intensity TUS may manipulate GABAergic neurotransmission in emergence from propofol anaesthesia; however, this process may be more complex [6]. Second, low-intensity TUS may partially affect GABAergic neurotransmission through indirect modulation of thalamo-cortical connectivity. The process might be analogous to anodal transcranial direct current stimulation over the motor cortex increasing the functional coupling between the thalamus and cortex and enhancing thalamic activity [30].

Previous studies have demonstrated that varying ultrasound parameters (frequency, intensity, pulse time, duty cycle) can achieve different neuromodulation effects. For example, ultrasound at lower frequencies (0.25–0.35 MHz) was more effective in evoking motor responses in anaesthetized mice [18]. The motor response and neural activity are closely related to ultrasound intensity. For example, the robustness of motor responses elicited by ultrasound stimulation increases with increasing ultrasound intensity [31]. The firing rate of neuronal action potentials evoked by ultrasound stimulation also increases with ultrasound intensity [32]. The magnitude of the LFPs and the coupling strength between the LFPs and haemodynamics increase linearly with the ultrasound intensity [33]. Based on the above findings, we speculate that the process of ultrasound stimulation to promote the recovery of anaesthetized animals may be affected by ultrasound parameters such as ultrasound intensity. In our future work, we will carry out research in this area to explore the relationship between anaesthesia emergence and ultrasound parameters.

We know that wake-up anaesthesia techniques are a necessary method to ensure functional monitoring and accurate localization of lesions and brain functional areas during neurosurgery. How to choose the appropriate wake-up anaesthesia method plays an extremely important role in reducing or preventing anaesthesia complications. Ultrasound stimulation serves as a noninvasive and promising brain stimulation tool to modulate brain activity and associated behavioural changes. Our research has found that ultrasound stimulation can shorten the wake-up time from anaesthesia, which will help the subject recover from anaesthesia to the awake state as soon as possible and reduce associated risks. However, studies of low-intensity TUS in humans either in diseased or healthy conditions are still in an early stage. Further studies are needed to elucidate which arousal pathways are responsible for the specific actions of low-intensity TUS in promoting the awakening and neurological recovery of mice.

There are a few limitations to this study. First, this study only compared the effect of low-intensity TUS on the recovery process of mice under a single anaesthetic drug and at the same dose. Second, this study only compared the effects of low-intensity TUS on the recovery of anaesthetized mice under single stimulation parameter. Third, we only found that low-intensity TUS can significantly shorten the recovery time of anaesthetized mice, and did not detect the effects of low-intensity TUS on the cognitive function of mice under emergence and recovery from anaesthesia.

## Conclusions

In summary, the mean absolute power, relative power and entropy of LFPs from the mouse motor cortex can be altered by propofol anaesthesia. low-intensity TUS can modulate the mean absolute power, relative power and entropy of LFPs from anaesthesia to wakefulness and has a positive effect on recovery from propofol anaesthesia in mice.

## Data Availability

The datasets used and/or analysed during the current study are available from the corresponding author upon reasonable request.
